# Linc-KIAA1737–2 promoted LPS-induced HK-2 cell apoptosis by regulating miR-27a-3p/TLR4/NF-κB axis

**DOI:** 10.1007/s10863-021-09897-1

**Published:** 2021-06-02

**Authors:** Ming Hu, Jing Wei, Liu Yang, Jianhua Xu, Zhaofeng He, Haiyuan Li, Chao Ning, Shijun Lu

**Affiliations:** 1 Department of Anesthesiology, Sir Run Run Shaw Hospital, Zhejiang University, School of Medicine, Hangzhou, China; 2 Department of Neurology, Chongqing Emergency Medical Center, The Affiliated Central Hospital to Chongqing University, Chongqing, China; 3 Department of Critical Care Medicine, Shandong Province Linyi Central Hospital, Linyi, Shandong China; 4 Department of General practice, Qingdao Ninth People’s Hospital, 2th Chaocheng Road, Qingdao, Shandong People’s Republic of China; 5 Department of Critical Care Medicine, Shandong Province Juxian People’s Hospital, Juxian, Shandong China

**Keywords:** Sepsis, Acute kidney injury, Linc-KIAA1737–2, miR-27a, TLR4, NF-κB

## Abstract

Inflammation and renal cell apoptosis participate in sepsis-induced acute kidney injury. Previous research found the upregulation of long non-coding RNA Linc-KIAA1737–2 in hypoxia- or inflammation-challenged human proximal tubular epithelial cells, but its role in sepsis-induced acute kidney injury is underexplored. In this research, we found that Linc-KIAA1737–2 could be upregulated in HK-2 human proximal tubular epithelial cells by LPS treatment, and knock-down of this lncRNA significantly attenuated LPS-induced apoptosis in HK-2 cells, while its overexpression showed opposite effect. MiR-27a-3p was confirmed to interact with Linc-KIAA1737–2 in HK-2 cells by RNA pull-down and dual-luciferase assay. MiR-27a-3p mimic transfection significantly attenuated LPS-induced HK-2 cell apoptosis by downregulating the protein levels of TLR4 and NF-κB, which was overturned by overexpression of Linc-KIAA1737–2. Our results suggested that Linc-KIAA1737–2 could promote LPS-induced apoptosis in HK-2 cells, and presumably sepsis-induced acute kidney injury, by regulating the miR-27a-3p/TLR4/NF-κB axis.

**Supplementary Information:**

The online version contains supplementary material available at 10.1007/s10863-021-09897-1.

## Introduction

Sepsis induced acute kidney injury.

Sepsis is a life-threatening symptom that can lead to dysfunction of lung, liver and kidney. From the physiological perspective, sepsis is often characterized as systemic inflammatory response syndrome (Hotchkiss et al. 2016). Epithelial cell apoptosis caused by inflammation and/or hypoxia is one of the major steps during the development of sepsis-induced acute kidney injury, which leads to kidney failure and patients’ poor prognosis (Lelubre and Vincent 2018). The inflammatory response in sepsis is initiated by the recognition of pathogen-associated molecular patterns, such as lipopolysaccharide (LPS), by pattern recognition receptors, principally Tool-like receptor 4 (TLR4), on the epithelial cell surface (Gay et al. 2014; Morrell et al. 2014). Activation of pattern recognition receptors induced the pro-inflammatory signaling through MAPK and NF-kappaB signaling pathway, which triggered or augmented the expression of genes involved in the innate immune response (Lelubre and Vincent 2018; Lawrence 2009). The inflammation causes epithelial cell apoptosis and increases epithelium permeability, resulting in tissue edema, anemia and hypoxia that aggravate the epithelial damage. Anti-inflammation treatment has thus been considered a possible approach to preventing or ameliorating kidney damage in septic patients (Bellomo et al. 2017; Poston and Koyner 2019).

It is known that long non-coding RNAs (lncRNAs) can regulate gene expression by interacting with microRNAs (miRNAs). While miRNAs regulate gene expression by interacting with mRNAs, preventing mRNA translation and mediating their degradation through the RNA-induced silencing complex; lncRNAs, on the other hand, competitively bind to miRNAs, thus inhibiting their functions (Beermann et al. 2016; Thomson and Dinger 2016). It has been found that various lncRNAs could promote or suppress the inflammatory response and related signaling pathways (Du et al. 2017; Hu et al. 2019; Liu et al. 2019), but research on the role of lncRNAs in the development of sepsis induced acute kidney injury is in its infancy. In a pilot study reported in 2015, Lin et al. found that three lncRNAs, namely MIR210HG, linc-ATP13A4–8, and linc-KIAA1737–2, were upregulated in human proximal tubular epithelial cells after challenged with hypoxia, pro-inflammatory cytokines or plasma from severe septic patients, proposing that these lncRNAs might be involved in the development of sepsis-induced acute kidney injury (Lin et al. 2015). Later in 2016, Chun-Mei et al. compared the landscape of lncRNA expression in serum between sepsis induced acute kidney injury patients and healthy counterparts; by mapping the predicted target genes of miRNAs presumably regulated by the differentially expressed LncRNAs to Kegg pathways, they proposed that lncRNA might participate in the development of sepsis-induced acute kidney injury by regulating the MAPK, PI3K-Akt, TGF-beta and NF-κB signaling pathways that were involved in the immune and inflammatory responses (Chun-Mei et al. 2016). PVT1 (Huang et al. 2017), NEAT1 (Chen et al. 2018; Jiang et al. 2019a), HOTAIR (Jiang et al. 2019b), TUG1 (Xu et al. 2020), TapSAKI (Shen et al. 2019) and XIST (Xu et al. 2019) were later found to either augment or weaken the inflammatory response in sepsis-induced acute kidney injury via miRNA sponging.

Treating renal epithelial cells with LPS is frequently employed to mimic cell injury during the development of Sepsis induced acute kidney injury (Pan et al. 2019; Nakano et al. 2015). In this research we used HK-2 cells to investigate the involvement of lncRNA Linc-KIAA1737–2 in sepsis-induced acute kidney injury. Our results showed that Linc-KIAA1737–2 promoted the LPS-induced apoptosis of HK-2 cells possibly by sponging and inhibiting miR-27a-3p, thus increasing the expression of TLR4 and NF-κB p65 subunit and augmenting the TLR4/NF-κB signaling pathway.

## Materials and methods

### HK-2 cells and LPS treatment

The inflammatory response and cell damage of LPS-induced apoptosis in HK-2 cells is a cellular model of acute kidney injury (AKI), which is one of the most fatal inflammatory sepsis (Huang et al. 2017; Shen et al. 2019; Zhang et al. 2020). Therefore, HK-2 cells were purchased from BeNa culture collection (Cat. No. BNCC100395, Beijing, China) and were cultured in RPMI-1640 medium (Procell, Wuhan, China) supplemented with 10% Fetal bovine serum (BeNa culture collection), 100 μg/mL penicillin, and 100 μg/mL streptomycin, incubated at 37 °C under 10% CO_2_ atmosphere. LPS (Sigma-Aldrich, Shanghai, China) was dissolved in RPMI-1640 medium at 1 mg/ml as the stock solution and was stored under −80 °C before use. At about 80% confluency, HK-2 cells were treated under cell culture condition with LPS either at 0, 1, 2, 5 or 10 μg/mL for 8 h or at 5 μg/mL for 2, 4, 8 or 10 h according to references (Huang et al. 2017; Li et al. 2017) before further analysis. The experiment was approved by the Shandong Province Linyi Central Hospital Ethics Committee.

### Cell transfection

Linc-KIAA1737–2 knock-down or overexpression in HK-2 cells was achieved by transfecting with siRNA or overexpression vectors, which were designed and constructed by RiboBio (Guangzhou, China). Two siRNAs for Linc-KIAA1737–2 (si-Linc-KIAA1737-2-1 and si-Linc-KIAA1737-2-2) were transfected using riboFECT™ CP Buffer (RiboBio) at a working concentration of 100 nM following manufacturer’s instructions; a non-targeting siRNA (si-NC, RiboBio) was used in parallel as negative control for Linc-KIAA1737–2 knock-down. Linc-KIAA1737–2 overexpression vector (pcDNA3.1(+)-Linc-KIAA1737–2) was transfected at a working concentration of 5 μg/mL using Lipofectamine 2000 reagent (Thermo Fisher Scientific, Shanghai, China) following manufacturer’s instructions; an empty vector (pcDNA3.1(+)) was used in parallel as negative control for Linc-KIAA1737–2 overexpression; cells with positive transfection were selected with neomycin. These cells were collected 72 h after transfection for further analysis.

MiR-27a-3p knock-down or overexpression was mimicked by transfection with two miR-27a-3p inhibitors (miR-27a-3p inhibitor-1 and miR-27a-3p inhibitor-2) or miR-27a-3p mimic, which were designed and constructed by RiboBio. MiRNA inhibitor and mimic with the same length and scrambled sequence were used as negative control for miR-27a-3p inhibitor and miR-27a-3p mimic, respectively. These small nucleotides were transfected using riboFECT™CP Reagent (RiboBio) at a working concentration of 200 nM following manufacturer’s instructions. Cells were collected 72 h after transfection for further analysis.

### QPCR analysis of Linc-KIAA1737–2 or miR-27a-3p level

Linc-KIAA1737–2 or miR-27a-3p level in HK-2 cells was evaluated by using customized BlazeTaq™ SYBR Green One-Step qPCR Kit or All-in-One™ miRNA qRT-PCR Detection Kit, respectively, which were provided by Genecopoeia (Guangzhou, China). Total RNA from HK-2 cells was first extracted using TRIzol reagent (Thermo Fisher Scientific) according to the manufacturer’s protocol. Nano-Drop-2000 nucleic acid analyzer was used to detect the concentration and purity of RNA to confirm that the A260/A280 ratio according to the MIQE guidelines (Bustin et al. 2009) of all samples was between 1.9–2.1. First-strand cDNA was reverse transcribed from RNAs using reverse transcriptase kit (Takara, Otsu, Japan), and 100 ng of total RNA was used as template for qPCR. The level of miR-27a-3p was normalized to that of U6 and GAPDH were used as internal reference genes for Linc-KIAA1737–2. As for the primers used, the amplification efficiency (E) was calculated according to the formula: E = (10^–1/slope^-1) × 100%. Primers with amplification efficiency between 90% and 110% met the requirements (Supplementary data, Draw1). qPCR result was expressed as fold-change comparing to the control group using 2^-ΔΔCq^ method. Each sample is divided into three multiple wells for testing. The sequence of primers for Linc-KIAA1737–2, miR-27a-3p, GAPDH and U6 were as followed:

Linc-KIAA1737–2:

5’-TACGGCCAAGACCATAGACC-3′ (forward);

5’-CTTCCCTCATGGGACAAAAA-3′ (reverse);

miR-27a-3p:

5’-TCCCGAATCGACGAACACTCGT-3′ (forward);

5’-GCGCGCGTAACAGTCTACAGC-3′ (reverse);

GAPDH:

5’-TGCACCACCAACTGCTTAGC-3′ (forward),

5’-GGCATGCACTGTGGTCATGAG-3′ (reverse);

U6:

5’-CTCGCTTCGGCAGCACA-3′ (forward),

5’-AACGCTTCACGAATTTGCGT-3′ (reverse);

### Flow cytometry evaluating cell apoptosis

Cell apoptosis was evaluated by flow cytometry using Annexin V-FITC cell apoptosis assay kit (Beyotime, Shanghai, China). HK-2 cells after treatment with 5 μg/ml of LPS for 8 h were collected using trypsin-EDTA (Thermo Fisher Scientific) and brief centrifugation (1000×g for 5 min at room temperature). 100,000 cells resuspended in PBS were then collected by centrifugation and were subject to Annexin V-FITC/PI double staining following manufacturer’s instructions. Cells with Annexin V-FITC^+^ PI^−^ staining were considered apoptotic cells (Schutte et al. 1998). The apoptotic result is calculated as Q2 + Q3, where Q2 is early cell apoptosis, and Q3 is late apoptosis. Our figures are drawn based on adopting the approach to calculating the percentage of Q2 + Q3.

### Western blotting assay

Protein level of Bcl-2, Bax, Cytochrome C (Ctyc), caspase 3, cleaved-caspase 3, caspase 9, cleaved-caspase9, TLR4, NF-κB or GAPDH were evaluated using antibodies purchased from Abcam (Shanghai, China). Cells were lysed using harsh RIPA lysis buffer (Betotime) and sonication, and total protein concentration of the cell lysate was measured by enhanced BCA protein concentration determination kit (Betotime). 30 ng of total proteins were then subject to western blotting. Primary antibodies were used at a working concentration of 0.5 to 1 μg/mL. GAPDH protein was used as internal reference.

### Nuclear/cytosol fractionation and fluorescence in situ hybridization (FISH) assay

Cellular fractionation and qPCR analysis of Linc-KIAA1737–2 in the cytosolic and nuclear fraction of HK-2 cells were performed by RiboBio. According to the service provider, HK-2 cells were fractionated by lysis using specific lysates of different strength according to the difficulty of cell membrane and nuclear membrane lysis. Linc-KIAA1737–2 levels in the cytosolic RNA and nuclear RNA were determined by qPCR using GAPDH as internal references gene of Linc-KIAA1737–2 and U6 as internal references gene of miR-27a-3p, respectively.

A customized Linc-KIAA1737–2 FISH probing kit (RiboBio) was used to localize Linc-KIAA1737–2 in HK-2 cells cultured on glass coverslips following manufacturer’s instructions with modifications. Briefly, cells were fixed with 4% paraformaldehyde and washed with PBS, before they were incubated with cold 0.5% Triton X-100 in PBS for 5 min at 37 °C. The coverslips were then blocked with pre-hybridization buffer associated with the kit for 30 min at 37 °C, after which the cells were incubated with fluorescent probe mix for 24 h at 37 °C. DAPI was then used for nuclear staining. After incubation, the cells were gently washed and fixed with mounting media (Thermo Fisher Scientific), and they were subject to observation by confocal microscopy.

### RNA-pull-down and dual-luciferase assay

RNA pull-down from total cell lysis was performed using biotin-labeled antisense probe against Linc-KIAA1737–2 constructed by RiboBio. HK-2 cells were lysed on ice with mild RIPA lysis buffer (Beyotime) and sonication. After centrifugation at 10000×g for 5 min, the supernatant was incubated with either the Linc-KIAA1737–2-capturing probe or a scrambled control probe at for 2 h at 4 °C, before the mixture was further incubated with streptavidin-conjugated agarose beads (Sigma-Aldrich) for 2 h at 4 °C. After brief centrifugation, RNAs associated with the beads were subject to qPCR analysis.

Dual-luciferase assay was performed using a customized pmirGLO Dual-Luciferase miRNA Target Expression Vector provided by Promega (Beijijg, China). Briefly, the Linc-KIAA1737–2-WT vector carried the firefly luciferase gene governed by a 3′ untranslated region carrying the wildtype miR-27a-3p-binding site on Linc-KIAA1737–2, and the Linc-KIAA1737–2-MUT vector carried the miR-27a-3p-binding site on Linc-KIAA1737–2 at the 3′ untranslated region downstream of firefly luciferase gene. The vectors were transfected into HK-2 cells at a final concentration of 5 ng/μL together with either miR-27a-3p mimic or scrambled miRNA mimic negative control at the final concentration of 20 nM using Lipofectamine 2000 described above. After 48 h of transfection, the firefly and Renilla luciferase activity was measured using the Dual-Glo Luciferase assay system (Promega) and a plate reader following manufacturer’s instructions. The activity of the firefly luciferase activity was first normalized to that of Renilla luciferase from the same cell before analysis.

### Statistical analysis

Statistics analysis was performed using GraphPad Prism software (Ver. 8.3, GraphPad Software, San Diego, CA, USA). Each group of data in the histograms was presented as mean ± SD; All the experiments were performed three times repeatedly(*n* = 3), and all data in each histogram were normalized to the mean value of the control group and were presented as fold change, unless otherwise indicated. Comparison between two groups of data was performed by Student’s t test. Multi-group comparisons were performed by One-way analysis of variance with Tukey’s test as the post-hoc test. A difference was considered statistically significant when *p* < 0.05.

## Results

### Linc-KIAA1737–2 is upregulated in LPS-challenged HK-2 cells

**Fig. 1:**
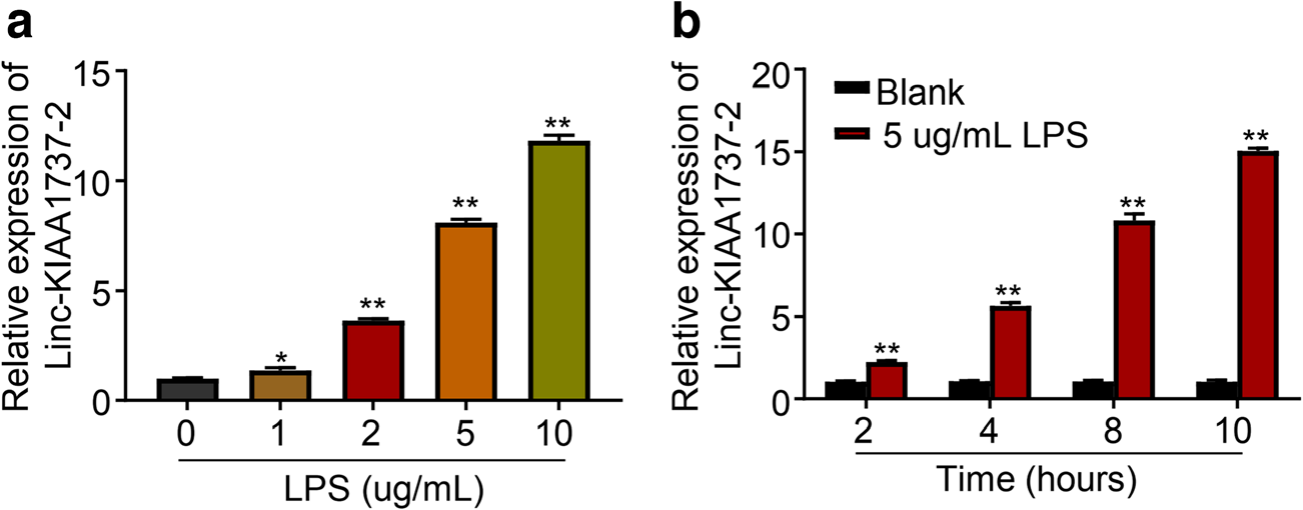
**LPS treatment upregulated Linc-KIAA1737–2 in HK-2 cells. A**. Cells were treated with LPS at indicated concentration for 8 h; **B**. Cells were treated with LPS at 5 μg/mL for indicated hours. Cells after these treatments were collected for qPCR analysis. *p < 0.05; **p < 0.01

LncRNA Linc-KIAA1737–2 has been found upregulated in hypoxia or pro-inflammatory cytokine-challenged proximal tubular epithelial cells (Lin et al. 2015). The role of this lncRNA in Sepsis induced acute kidney injury, however, remained to be unexplored. In this research, we first examined whether the expression level of Linc-KIAA1737–2 in renal epithelial cells would be influenced by LPS challenge. As shown in Fig. 1, the level of lncRNA Linc-KIAA1737–2 in HK-2 cells was drastically upregulated by LPS treatment in unequal dose at the same time. Interestingly, compared to that in HK-2 cells treated with vehicle in parallel, time-dependent manners was followed in 5μg/ml dose of Linc-KIAA1737–2 level.

### Linc-KIAA1737–2 facilitated HK-2 cell apoptosis

**Fig. 2:**
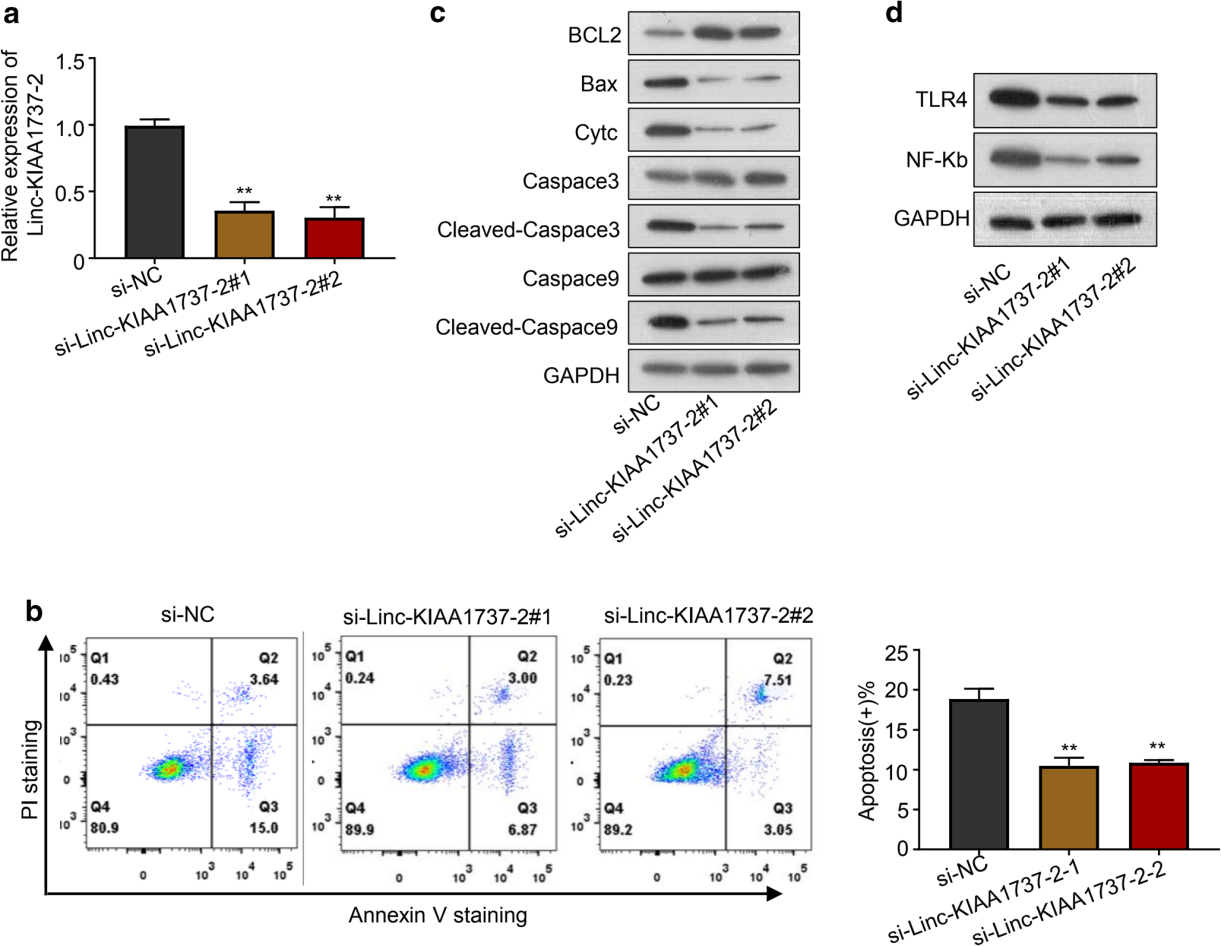
**Linc-KIAA1737–2 knock-down inhibited LPS (5 μg/mL)-induced HK-2 cell apoptosis. A**. Linc-KIAA1737–2 knock-down efficiency was verified by qPCR analysis after siRNA transfection. **B**, **C**. HK-2 cells with indicated transfection were treated with LPS (5 μg/mL), before they were subject to B. flow cytometry to evaluate cell apoptosis (Q1: Annexin V-, PI+ cells; Q2: Annexin V+, PI+ cells; Q3: Annexin V+/PI- cells; Q4: Annexin V-, PI- cells). **C**. The expression about pro-apoptotic protein, including Bax, Cytc, Caspace3 and Caspace9 as well as anti-apoptotic protein BCL2 were detected by western blotting to evaluate cell apoptosis. **D**. After LPS (5 μg/mL) treatment, HK-2 cells with indicated transfection were subject to western blotting to evaluate TLR4 and NF-κB p65 subunit protein levels. **p < 0.01

**Fig. 3:**
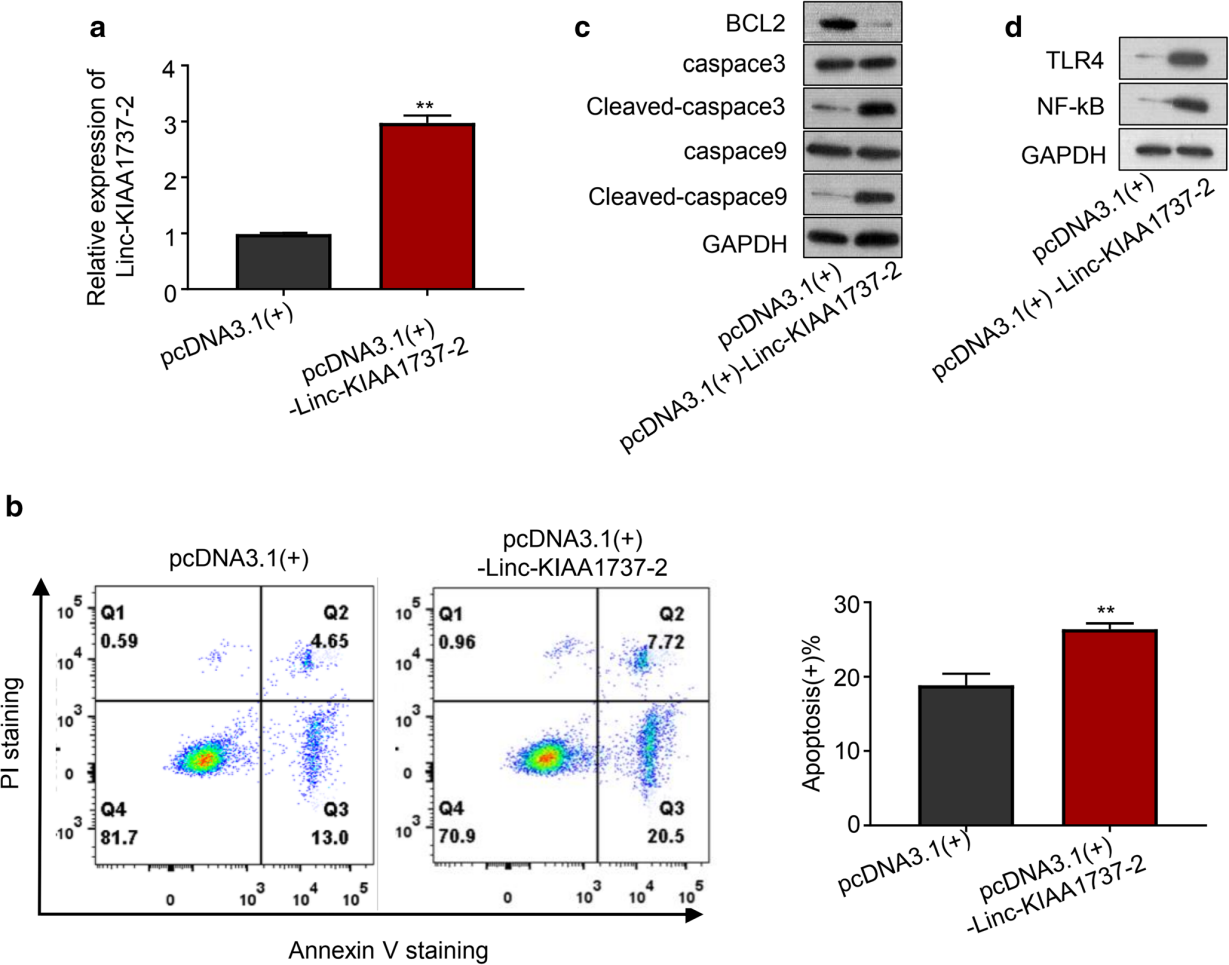
**Overexpression of Linc-KIAA1737–2 promoted LPS (5** μg**/mL)-induced HK-2 cell apoptosis. A**. Overexpression of Linc-KIAA1737–2 in HK-2 cells after transduction was verified by qPCR analysis. **B**, HK-2 cells with indicated transduction were treated with LPS (5 μg/mL) as indicated in Fig. 2, before they were subject to flow cytometry assay to evaluate cell apoptosis (Q1: Annexin V-, PI+ cells; Q2: Annexin V+, PI+ cells; Q3: Annexin V+/PI- cells; Q4: Annexin V-, PI- cells). **C**. The expression about pro-apoptotic protein, including Caspace3 and Caspace9 as well as anti-apoptotic protein BCL2 were detected by western blotting to evaluate cell apoptosis. **D**. After LPS (5 μg/mL) treatment, HK-2 cells with indicated transduction were subject to western blotting to evaluate TLR4 and NF-κB p65 subunit protein levels. **p < 0.01

Cell apoptosis is involved in the development of sepsis-induced acute kidney injury, while the inhibition of renal cell apoptosis has been proposed to alleviate this critical illness (Havasi and Borkan 2011; Wan et al. 2008). To investigate whether Linc-KIAA1737–2 participated in sepsis-induced acute kidney injury, we evaluated the impact of Linc-KIAA1737–2 knock-down on the LPS-induced apoptosis of HK-2 cells (si-Linc-KIAA1737–2#1, si-Linc-KIAA1737–2#2). As shown in Fig. 2A, qPCR analysis results confirmed over 50% reduction of this lncRNA’s level in HK-2 cells after knock-down by siRNA transfection; In Fig. 2B (early apoptosis: Annexin V+/PI-, late apoptotic/early necrotic: Annexin V+, PI+ cells, and late necrotic: Annexin V-, PI+ cells), Linc-KIAA1737–2 knock-down was significantly reduced LPS induced-HK-2 cell apoptosis, and this discovery verified by western-blotting analysis results (caspace3 and caspace9 are typical pro-apoptotic factors) (Fig. 2C). On the other hand, overexpression of this lncRNA significantly enhanced LPS-induced apoptosis of HK-2 cells, as suggested by our qPCR, western blotting and flow cytometry analysis results presented in Fig. 3. These data revealed the pro-apoptotic role of this lncRNA in renal tubular epithelial cells. Notably, we found that Linc-KIAA1737–2 knock-down significantly reduced the protein levels of TLR4 and NF-κB in LPS induced-HK-2 cells, while the overexpression of this lncRNA showed opposite effects, as suggested by our western blotting assay results shown in Fig. 2D and 3D.

### Linc-KIAA1737–2 promoted LPS induced-HK-2 cell apoptosis by sponging miR-27a-3p

**Fig. 4:**
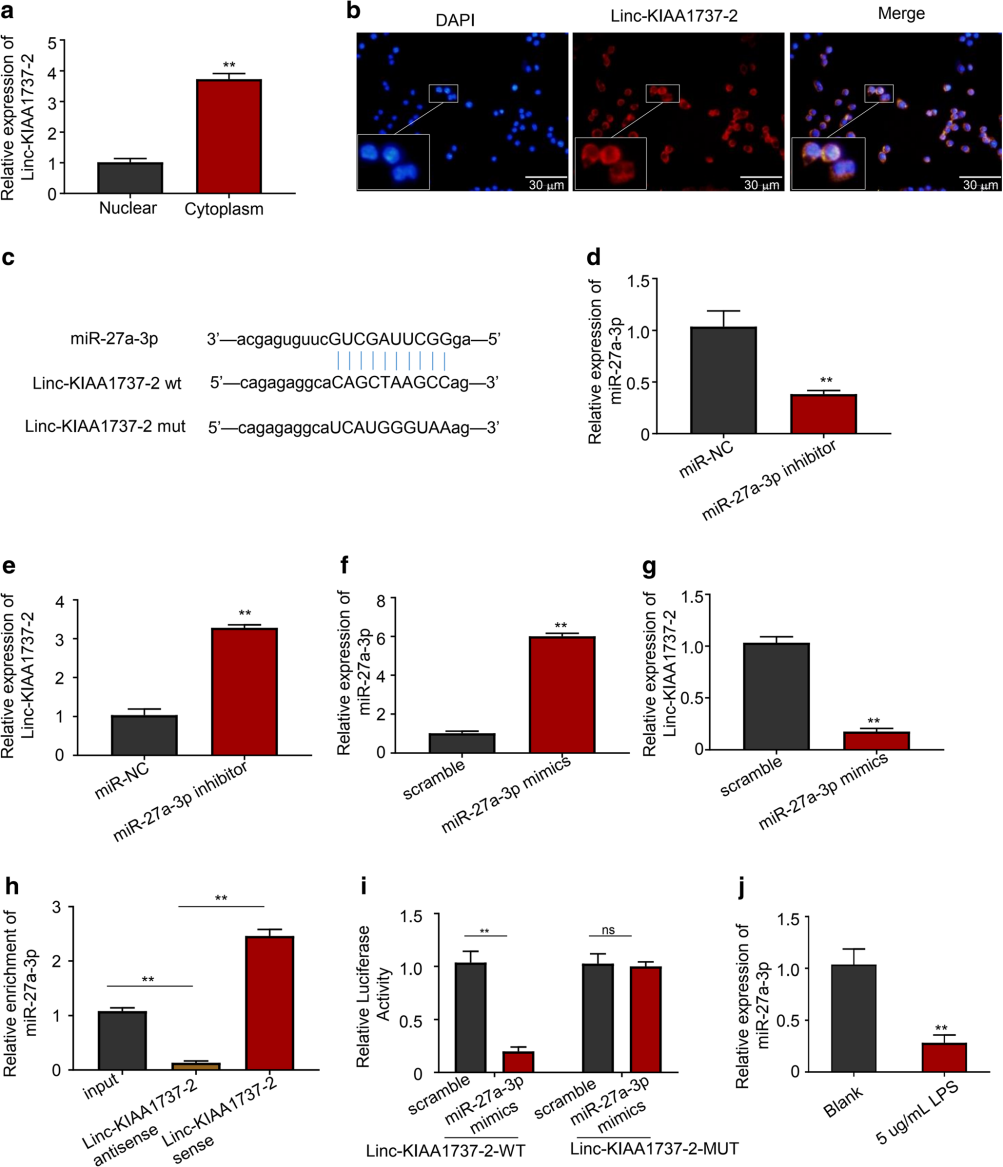
**Linc-KIAA1737–2 interact with miR-27a-3p in HK-2 cells. A**, **B**. Cytoplasmic localization of Linc-KIAA1737–2 was confirmed by nuclear/cytosol fractionation-associated qPCR and fluorescence in situ hybridization. **C**. Interaction between Linc-KIAA1737–2 and miR-27a-3p with the binding site was predicted by bioinformatic analysis. **D**, **E**. Transfection with miR-27a-3p inhibitor significantly increased Linc-KIAA1737–2 level in HK-2 cells. **F**, **G**. Transfection with miR-27a-3p mimics significantly decreased Linc-KIAA1737–2 level in HK-2 cells. **H**. RNA pull-down using Linc-KIAA1737–2-targeting antisense probe enriched miR-27a-3p from cell lysate. **I**. Interaction between Linc-KIAA1737–2 and miR-27a-3p as well as the binding site presented were verified by dual-luciferase assay. **J**. MiR-27a-3p levels in HK-2 cells after LPS (5 μg/mL) -induced or not was evaluated by qPCR analysis. ^**^p < 0.01; ns, not significant

The pathway of Linc-KIAA1737–2 hasn’t been explored before. By qPCR and FISH assay, we confirmed that this lncRNA primarily localized in the cytoplasm in HK-2 cells, as shown in Fig. 4A and B. Our bioinformatic analysis results further indicated that miR-27a-3p might interact with Linc-KIAA1737–2, as shown in Fig. 4C. It has been found that miR-27a-3p can alleviate hypoxia-induced renal tubular epithelial apoptosis (Jiang et al. 2019a). To verify the interaction between Linc-KIAA1737–2 and miR-27a-3p, we transfected LPS induced-HK-2 cells with miR-27a-3p inhibitor or mimics and analyzed their effect on the expression level of Linc-KIAA1737–2. As shown in Fig. 4D-4G, our qPCR analysis results disclosed that transfection with miR-27a-3p inhibitors significantly reduced the level of miR-27a-3p and increased that of Linc-KIAA1737–2, while transfection with the mimics of this miRNA significantly reduced Linc-KIAA1737–2 level. We further performed RNA pull-down assay by using Linc-KIAA1737–2-capturing antisense probe or scrambled probe as negative control, and found that pull-down of this lncRNA could enrich miR-27a-3p from total cell lysate, as shown in Fig. 4H, indicating that Linc-KIAA1737–2 can interact with miR-27a-3p to regulated the expression of target mRNA. To further justify the interaction between Linc-KIAA1737–2 and miR-27a-3p, we performed dual luciferase assay and verified the binding site of miR-27a-3p on Linc-KIAA1737–2. As shown in Fig. 4I, co-transfection with miR-27a-3p mimic with Linc-KIAA1737–2-WT vector significantly reduced the activity of firefly luciferase in LPS induced-HK-2 cells; co-transfection with the scrambled miRNA mimic negative control had a minimal effect on the firefly luciferase activity in LPS induced-HK-2 cells transfected with Linc-KIAA1737–2-WT, while the firefly luciferase activity in LPS induced-HK-2 cells transfected with Linc-KIAA1737–2-MUT was not affected by miR-27a-3p mimic transfection. Finally, we detected the expression level of miR-27a-3p in LPS-induced HK-2 cells (Fig. 4J), the results showed that compared with the expression in uninduced cells, the expression level of miR-27a-3p in LPS-induced cells was significantly reduced, and combined with other experimental results, it can be seen that the expression of miR-27a-3p and Linc-KIAA1737–2 were negatively correlated in HK-2 cells. The role of lncRNA sponging miRNA has been reported in many literatures (Zhang et al. 2018a; Dori and Bicciato 2019; Zhu et al. 2019), which can also justify our results.

### Linc-KIAA1737–2 promoted LPS-induced HK-2 cell apoptosis by suppressing the function of miR-27a-3p

**Fig. 5:**
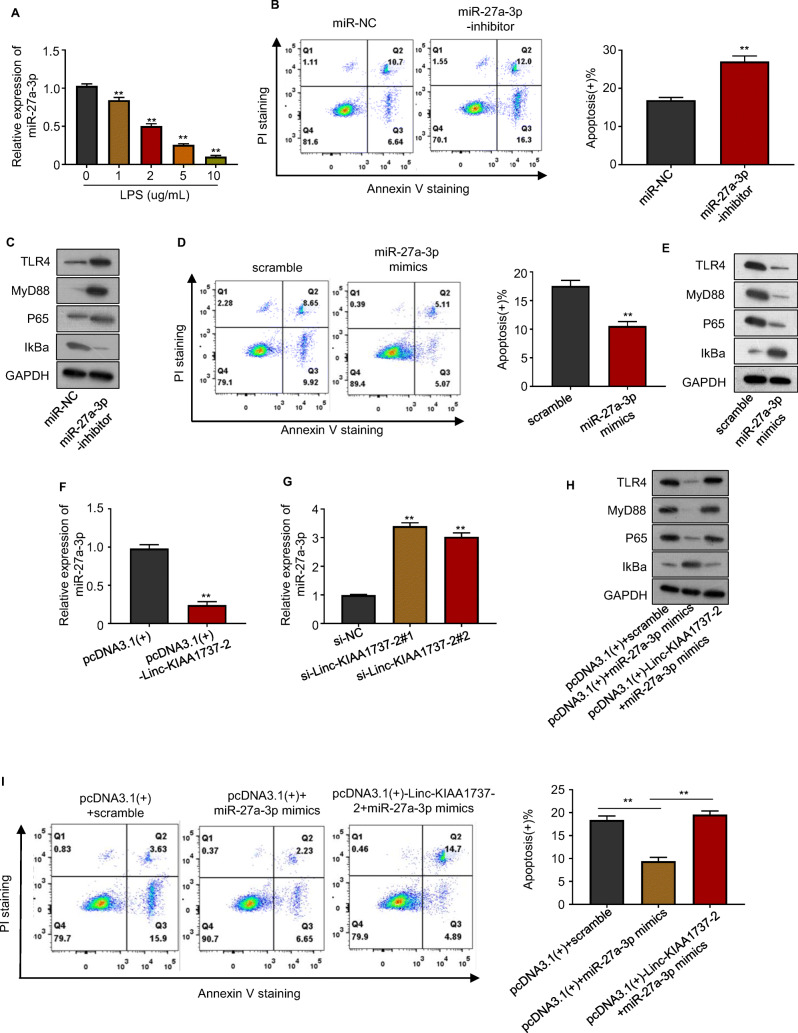
**Linc-KIAA1737–2 inhibited miR-27a-3p to augment TLR4/NF-κB signaling and LPS (5 μg/mL) -induced HK-2 cell apoptosis**. **A**. MiR-27a-3p levels in HK-2 cells after indicated treatment were evaluated by qPCR. **B**, **D**. LPS (5 μg/mL) -induced apoptosis of HK-2 cells with indicated transfection was evaluated by flow cytometry (Q1: Annexin V-, PI+ cells; Q2: Annexin V+, PI+ cells; Q3: Annexin V+/PI- cells; Q4: Annexin V-, PI- cells). **C**, **E**. Protein levels of TLR4, MyD88, NF-κB p65 subunit and IκBα in LPS (5 μg/mL) -treated HK-2 cells with indicated transfection were evaluated by western blotting assay. **F**, **G**. MiR-27a-3p levels in HK-2 cells after Linc-KIAA1737–2 overexpression or knock-down was evaluated by qPCR analysis. **I**. HK-2 cells with or without Linc-KIAA1737–2 overexpression was transfected with miR-27a-3p mimic, before they were subject to LPS (5 μg/mL) treatment and flow cytometry analysis to evaluate cell apoptosis. **H**. HK-2 cells after indicated transfection were subject to western blotting analysis to evaluate the protein levels of TLR4, MyD88, NF-κB p65 subunit and IκBα. **p < 0.01

MiR-27a-3p has previously been found to protect mice lung and liver from septic injury by reducing the expression of NF-κB p65 subunit and TLR4 (Yang et al. 2018; Ju et al. 2018). Based on these results, we proposed that Linc-KIAA1737–2 might facilitate LPS-induced HK-2 cell apoptosis by sponging miR-27a-3p, thus inhibiting the function of this miRNA. We first found that LPS treatment could significantly reduce the level of miR-27a-3p in HK-2 cells in a dose-dependent manner, as demonstrated by our qPCR assay results shown in Fig. 5A, then we found out that transfection of miR-27a-3p inhibitor significantly increased the LPS-induced apoptosis in HK-2 cells by flow cytometry analysis, as shown in Fig. 5B, which confirmed the anti-apoptotic role of this miRNA. Western blotting assay results manifested that transfection with miR-27a-3p inhibitor significantly upregulated the protein levels of TLR4, MyD88, NF-κB p65 subunit, while reducing that of IκBα in HK-2 cells (Fig. 5C). It suggested that miR-27a-3p might inhibit the LPS-induced inflammatory response in HK-2 cells by downregulating the TRL4- NF-κB signaling. These results were further verified by flow cytometry (Fig. 5D) and western blotting analysis (Fig. 5E) using miR-27a-3p mimic. Our qPCR assay results further presented that overexpression of Linc-KIAA1737–2 significantly reduced the level of miR-27a-3p in HK-2 cells, while the knock-down of this lncRNA showed opposite effect, as presented in Fig. 5F and G; overexpression of Linc-KIAA1737–2 overturned the apoptosis-reducing and TRL4-NF-κB signaling-inhibiting effect of miR-27a-3p mimic transfection in LPS induced HK-2 cells, as clarified by our flow cytometry (Fig. 5H) and western blotting analysis (Fig. 5I) results. These results expounded that Linc-KIAA1737–2 could facilitate LPS-induced HK-2 cell apoptosis by sponging miR-27a-3p, thus suppressing the function of this miRNA.

## Discussion

Cell apoptosis caused by inflammation or hypoxia participates in the development of sepsis-induced acute kidney injury. In a pilot research, Lin et al. found that three LncRNAs mostly upregulated by hypoxia or pro-inflammatory cytokine challenge in human proximal tubular epithelial cells, including MIR210HG, linc-ATP13A4–8 and linc-KIAA1737–2; they further discovered that treating viable human proximal tubular epithelial cells with septic patients’ plasma also elevated the level of linc-ATP13A4–8 and linc-KIAA1737–2 in these cells (Lin et al. 2015). These findings represented that linc-ATP13A4–8 and linc-KIAA1737–2 might be involved in the development of sepsis-induced acute kidney injury, and the function of two lncRNAs haven’t been studied before. In this research, we chose linc-KIAA1737–2 for analysis, but linc-ATP13A4–8 may also deserve to be investigated.

We identified that Linc-KIAA1737–2 could be upregulated in HK-2 human proximal tubular epithelial cells by LPS treatment; knock-down of this lncRNA significantly attenuated LPS-induced apoptosis in HK-2 cells, while its overexpression showed opposite effect, suggesting the participation of this lncRNA in sepsis-induced acute kidney injury. The TLR4-NF-κB axis plays a central part in the inflammatory response and apoptosis of renal epithelial cells during sepsis-induced acute kidney injury (Morrell et al. 2014; Anderberg et al. 2017; Gao et al. 2017). Therefore, we examined the protein levels of TLR4 and NF-κB p65 subunit in LPS-induced HK-2 cells with or without Linc-KIAA1737–2 overexpression/knock-down. We uncovered that knock-down of this lncRNA significantly reduced the protein level of TLR4 and NF-κB p65 subunit, while its overexpression showed opposite effect, indicating that Linc-KIAA1737–2 might facilitate LPS-induced HK-2 cell apoptosis as well as sepsis-induced acute kidney injury by upregulating the protein levels of TLR4 and NF-κB p65 subunit.

LncRNAs are known to regulate gene expression by sponging miRNAs and rescuing mRNA translation. Previous researchers have noticed that miR-27a-3p could repress inflammatory response by reducing TLR4 expression via direct mRNA targeting in various types of cells (Ju et al. 2018; Zhang et al. 2018b; Lv et al. 2017); this miRNA was also found to reduce the protein levels of NF-κB p65 and MyD88 that were elevated in LPS-challenged RAW264.7 cells. It proclaimed that miR-27a-3p might alleviate sepsis-induced acute lung injury (Ju et al. 2018). Using miRanda software we found that Linc-KIAA1737–2 might interact with miR-27a-3p, and we hypothesized that Linc-KIAA1737–2 might facilitate LPS-induced HK-2 cell apoptosis as well as sepsis-induced acute kidney injury by sponging and inhibiting miR-27a-3p. We confirmed the interaction between miR-27a-3p and Linc-KIAA1737–2 in HK-2 cells by RNA pull-down and dual- luciferase assay. MiR-27a-3p mimic transfection significantly attenuated LPS-induced HK-2 cell apoptosis by downregulating the protein levels of TLR4 and NF-κB, which was overturned by overexpression of Linc-KIAA1737–2. Notably, we also corroborated that Linc-KIAA1737–2 primarily localize in the cytosol, which is consistent with Lin et al’s results (Lin et al. 2015).

A major drawback of this research is a lack of in vivo investigation. We believed that a significant difference in the level of Linc-KIAA1737–2 should be found in kidney tissues between viable and septic mice, and knock-down of this lncRNA might ameliorate sepsis-induced acute kidney injury in vivo. These perceptions will be examined in our future research.

## Conclusions

In this research, for the first time we have found that Linc-KIAA1737–2 promoted LPS-induced apoptosis in human proximal tubular epithelial cells by regulating the miR-27a-3p/TLR4/NF-κB axis. This lncRNA might be used as a diagnostic or prognostic biomarker for sepsis-induced acute kidney injury, and targeting this lncRNA for treatment of this critical illness deserved further investigation.

### Data available on request owing to privacy/ethical restrictions

All supporting data of this work, which are available from the corresponding author upon request. The e-mail is: lushijun2020@126.com.

## Supplementary Information

ESM 1 (DOCX 605 kb)

ESM 2 (PNG 14 kb)
