# Supplementary material for: Linc-KIAA1737–2 promoted LPS-induced HK-2 cell apoptosis by regulating miR-27a-3p/TLR4/NF-κB axis
Source: J Bioenerg Biomembr. 2021 Jun 2;53(4):393–403. doi: 10.1007/s10863-021-09897-1 (PMC8360891; doi:10.1007/s10863-021-09897-1)
Supplement: Supplementary file 1 — (DOCX 605 kb) [file 10863_2021_9897_MOESM1_ESM.docx]

**Supplementary Data:**

**
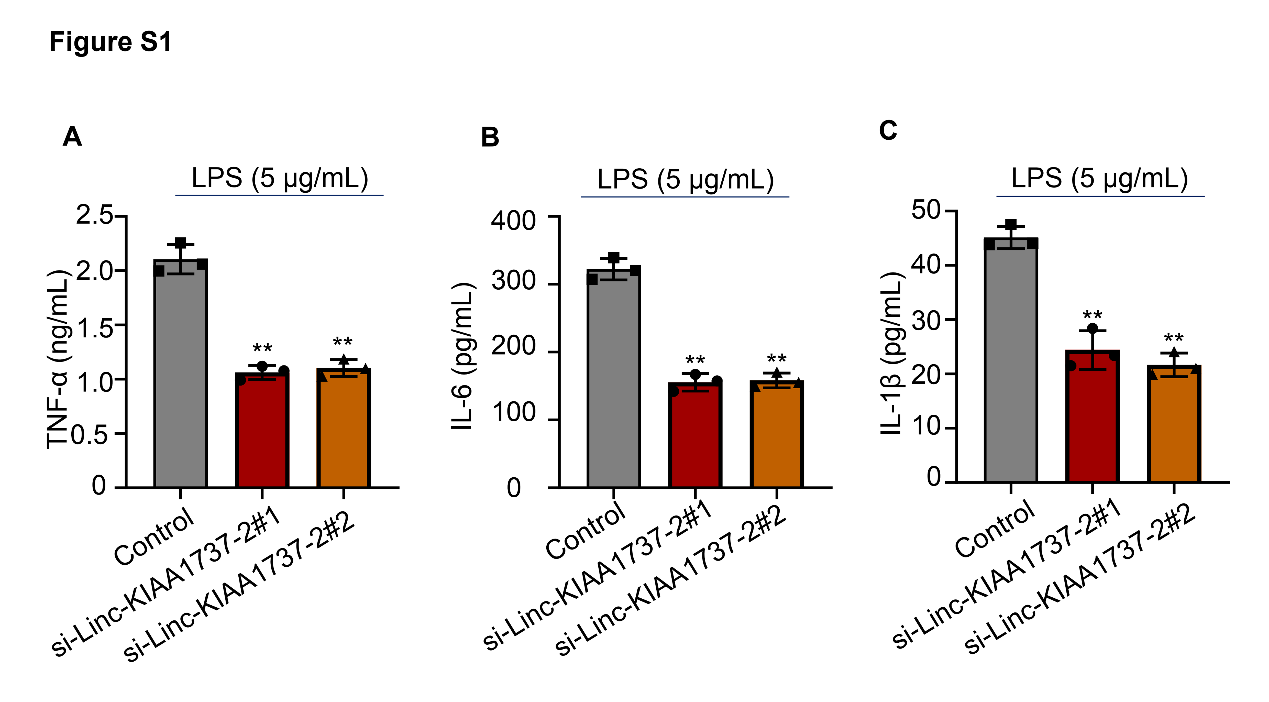
**

**Fig S1.** The expression level of TNF-α, IL-6, and IL-1β were significantly downregulated after Linc-KIAA1737-2 silencing.

**
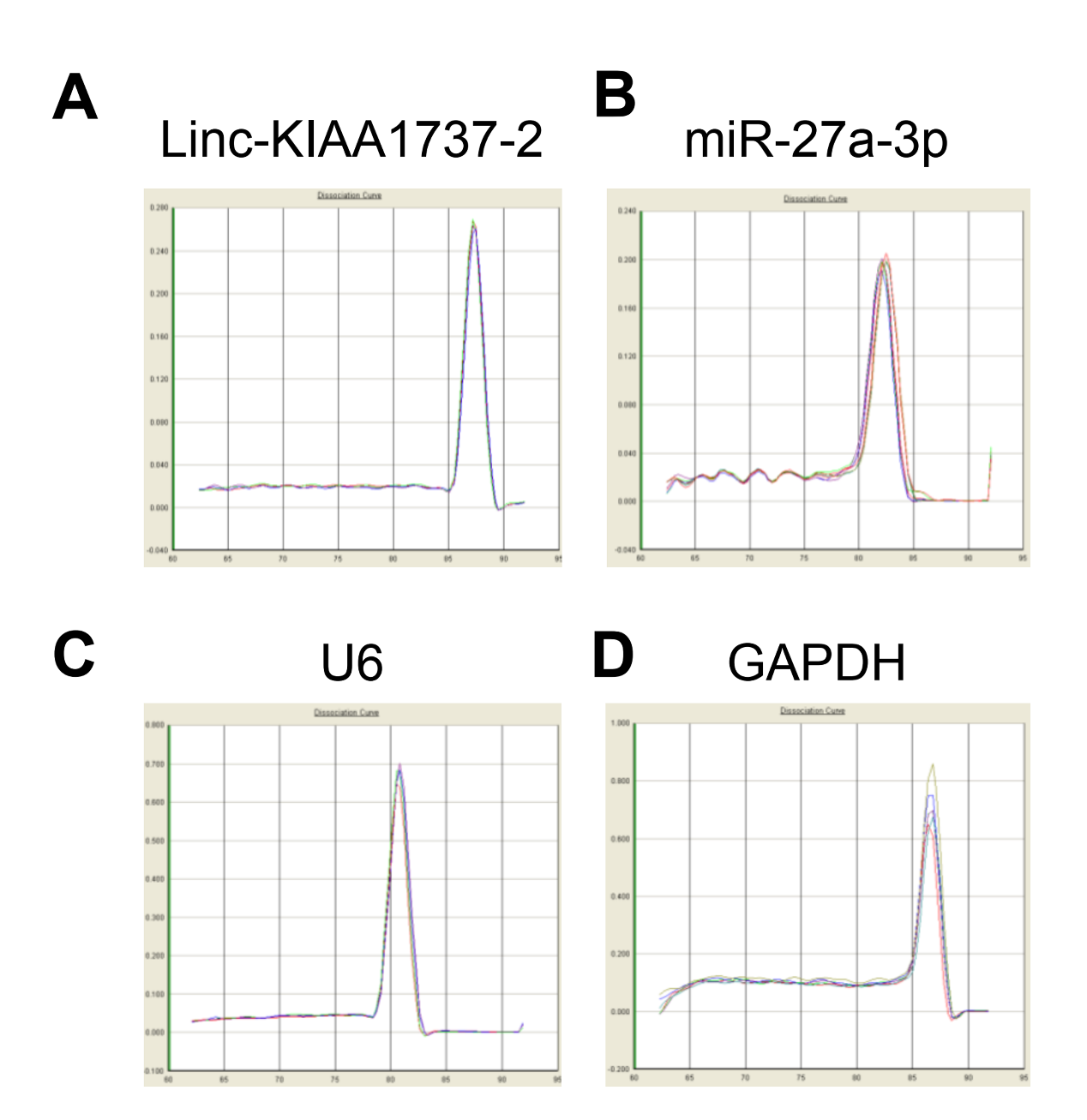
**

**Fig S2. Melting curve of reference genes.** (A) The melt curves of Linc-KIAA1737-2. (B) miR-27a-3p. (C) U6. (D) GAPDH.

**Table S1. Amplification characteristics of the reference genes in qRT-PCR.**

| **Gene** | **Efficiency (%)** | **Correlation coefficient (R^2^)** | **Slope** |
| --- | --- | --- | --- |
| Linc-KIAA1737-2 | 97.70 | 0.998 | -3.343 |
| miR-27a-3p | 98.81 | 0.999 | -3.356 |
| GAPDH | 101.59 | 0.996 | -3.251 |
| U6 | 95.03 | 0.995 | -3.479 |
