# Supplementary figures and images for: Linc-KIAA1737–2 promoted LPS-induced HK-2 cell apoptosis by regulating miR-27a-3p/TLR4/NF-κB axis
Source: J Bioenerg Biomembr. 2021 Jun 2;53(4):393–403. doi: 10.1007/s10863-021-09897-1 (PMC8360891; doi:10.1007/s10863-021-09897-1)

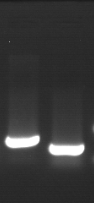

Supplement: Supplementary file 2 — (PNG 14 kb) [file 10863_2021_9897_MOESM2_ESM.png]
